# Dietary Phytochemicals: Natural Swords Combating Inflammation and Oxidation-Mediated Degenerative Diseases

**DOI:** 10.1155/2016/5137431

**Published:** 2016-09-19

**Authors:** Md. Asiful Islam, Fahmida Alam, Md. Solayman, Md. Ibrahim Khalil, Mohammad Amjad Kamal, Siew Hua Gan

**Affiliations:** ^1^Human Genome Centre, School of Medical Sciences, Universiti Sains Malaysia, 16150 Kubang Kerian, Kelantan, Malaysia; ^2^Department of Biochemistry and Molecular Biology, Jahangirnagar University, Savar, Dhaka 1342, Bangladesh; ^3^King Fahd Medical Research Center, King Abdulaziz University, P.O. Box 80216, Jeddah, Saudi Arabia; ^4^Enzymoics, 7 Peterlee Place, Hebersham, NSW 2770, Australia; ^5^Novel Global Community Educational Foundation, 7 Peterlee Place, Hebersham, NSW 2770, Australia

## Abstract

Cumulatively, degenerative disease is one of the most fatal groups of diseases, and it contributes to the mortality and poor quality of life in the world while increasing the economic burden of the sufferers. Oxidative stress and inflammation are the major pathogenic causes of degenerative diseases such as rheumatoid arthritis (RA), diabetes mellitus (DM), and cardiovascular disease (CVD). Although a number of synthetic medications are used to treat these diseases, none of the current regimens are completely safe. Phytochemicals (polyphenols, carotenoids, anthocyanins, alkaloids, glycosides, saponins, and terpenes) from natural products such as dietary fruits, vegetables, and spices are potential sources of alternative medications to attenuate the oxidative stress and inflammation associated with degenerative diseases. Based on* in vitro*,* in vivo*, and clinical trials, some of these active compounds have shown good promise for development into novel agents for treating RA, DM, and CVD by targeting oxidative stress and inflammation. In this review, phytochemicals from natural products with the potential of ameliorating degenerative disease involving the bone, metabolism, and the heart are described.

## 1. Introduction

Degenerative diseases occur due to the continuous deterioration of cells and tissues that ultimately affects major organs. Both oxidative stress and inflammation are considered major role players in the pathogenesis of chronic degenerative diseases including cardiovascular diseases (CVDs) [1], diabetes mellitus (DM) [2], and rheumatoid arthritis (RA) [3]. All chronic degenerative diseases exert an immense impact on the global health economy [4–6]. Currently, although several synthetic regimens are used to attenuate oxidative stress and inflammation-mediated degenerative diseases, none are free from side effects when utilized in the treatment of CVDs [7], DM [8], or RA [9].

Over the last two decades, tremendous experimental advancements have been made in the use of natural products against different types of degenerative diseases targeting oxidative stress and inflammation [10]. Many studies have also demonstrated that phytochemicals are important therapeutic agents targeting oxidative stress and inflammation, which are the major culprits in the pathogenesis of chronic degenerative diseases [11, 12]. Some of these phytochemicals are good candidates for future drug discovery and development to treat degenerative diseases [13–16].

In this review, we discussed the pathogenesis of CVDs, DM, and RA, which involve the heart, metabolism and the joints, respectively, and we discussed the use of phytochemicals (which are synthesized by fruits, vegetables, and spices) in attenuating the oxidative stress and inflammation associated with these chronic diseases.

## 2. Methodology

The PubMed database was systematically searched to retrieve evidence of potential dietary natural products (fruits, vegetables, and spices) and their active substances (antioxidants, polyphenols, carotenoids, anthocyanins, alkaloids, glycosides, saponins, and terpenes) for use against CVDs, DM, and RA by attenuating oxidative stress and inflammation (see Appendix). To retrieve clinical and experimental evidences of dietary phytochemicals associated with CVDs, DM, and RA, only papers published in English between January 2000 and March 2016 were considered.

## 3. Cardiovascular Diseases

CVDs are a group of diseases associated with complications of the heart and blood vessels; most are associated with coronary heart disease. Major risk factors of CVDs include hypertension (HTN), hypercholesterolemia, diabetes, obesity, inflammation, smoking, consumption of alcohol, lack of exercise, and a familial history of heart diseases [17, 18].

CVD is believed to be the major contributor to worldwide mortality and morbidity in both developed and developing countries. In 2012, it was estimated that 17.5 million people died from CVDs, which represents 31% of all global deaths. Among these deaths, it was estimated that 7.4 million were due to coronary heart disease. It has also been predicted that, by 2030, over 28 million people will die from CVDs [19]. Based on the American Heart Association Report (2016), 85.6 million American adults were afflicted with CVDs, and this number is anticipated to rise and add a greater economic burden to the overall health care system [4].

### 3.1. Pathogenesis

**Figure 1:**
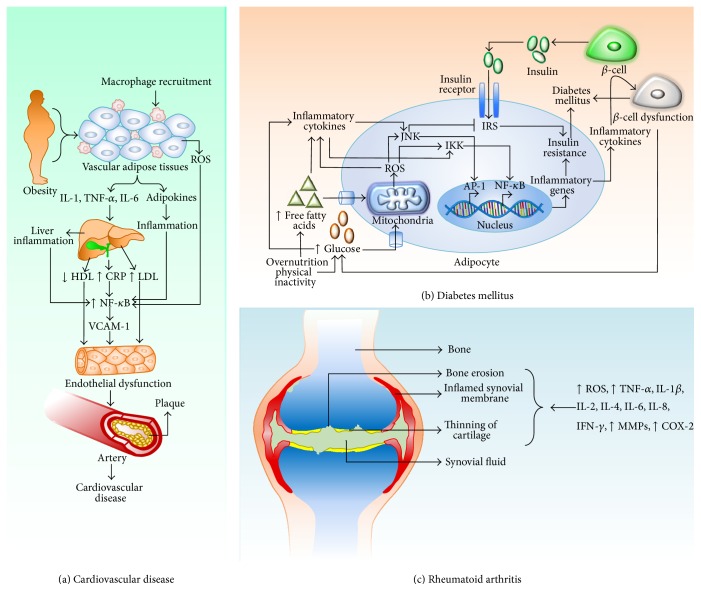
Inflammation and oxidative stress-mediated pathogenesis of (a) cardiovascular disease, (b) diabetes mellitus, and (c) rheumatoid arthritis.

The pathogenesis of CVDs is multifactorial, resulting from the interplay of genetic and environmental factors. However, atherosclerosis, which occurs due to the accumulation of atherosclerotic plaques within the walls of the arteries (Figure 1), is believed to be the major precursor of CVDs.

Plaque formation is initiated by endothelial damage and is followed by the adherence of circulating monocytes and subsequent exposure to homocysteine, inflammation, increased platelet aggregation, and higher levels of oxidized low density lipoprotein (LDL-ox) and reactive oxygen species (ROS) [20]. In addition, increased serum lipids such as triglycerides (TG) and cholesterol (C), increased plasma fibrinogen and coagulation factors, abnormal glucose metabolism, and hypertension play crucial roles in the pathogenesis of CVDs [21]. Adherent monocytes differentiate into macrophages, which ingest LDL-ox and transform into large foam cells, appearing as a fatty streak.

Additionally, although intact endothelium can prevent smooth muscle proliferation by releasing nitric oxide (NO) when the endothelium is damaged, smooth muscle proliferation and migration are observed from the tunica media into the tunica intima in response to damaged endothelial cell-secreted cytokines. This activity induces the formation of a fibrous capsule covering the fatty streak. Due to calcification in the smooth muscle cells and on the unstable fatty streak, plaque hardening occurs, which further blocks the coronary arteries [22, 23].

Further, genetic alterations can adversely promote the development of CVDs. Some examples include mutations or allelic variations of the renin-angiotensin pathway, endothelial NO synthase, coagulation factors, and fibrinogen, which can lead to the development of atherosclerosis [24]. In addition, diets that are high in saturated fat, trans-fats, and salt; diets that are low in fruits, vegetables, and fish; smoking; the consumption of tobacco; and insufficient physical activity are cardiovascular risk factors [25, 26]. High density lipoprotein (HDL) levels are negatively correlated with CVDs; therefore, the consumption of fiber-rich diets, including fruits and vegetables, the control of high blood pressure, proper regulation of lipid and lipoprotein metabolism, decreased platelet aggregation, and increased antioxidant status should limit the progress of CVDs.

### 3.2. The Role of Dietary Phytochemicals

Although there are several synthetic regimens available for treating CVDs, none are free of side effects and limitations (Table 1). Over the last two decades, researchers confirmed that the consumption of regular fresh fruits, vegetables, and spices has the potential to lower CVD risks by attenuating oxidative stress and inflammatory mediators. Here, we discuss some of the potential experimental and clinical evidence (Table 2) in favor of treating CVDs by supplementation with fruits, vegetables, and spices.

#### 3.2.1. Fruits

A population-based prospective cohort study from nine areas in Japan (77,891 male and female subjects aged 45–74 years) suggested that fruit consumption protects against the risk of CVDs [27]. A meta-analysis by Wang et al. [28] provided evidence that a higher consumption of fruits and vegetables correlated with a lower risk of all-cause mortality, predominantly cardiovascular mortality. A cross-sectional study on Hispanic (*n* = 445) and non-Hispanic white elders (*n* = 154) found that the high-frequency consumption of fruits and vegetables lowered plasma C-reactive protein (CRP) and homocysteine concentrations, consequently reducing inflammation, which is considered the major risk factor of CVDs [29]. Recently (2016), a study on a Chinese population (512,891 adults ranging from 30 to 79 years old) from 10 diverse localities also revealed that a high level of fruit consumption was associated with decreased HTN and blood glucose levels, which significantly decrease the risks of CVDs [30]. Another study by van't Veer et al. [31] on a Dutch population reported that cardiovascular deaths could be reduced by 16% (approximately 8,000 deaths per year), ranging from 6% to 22%, through high intake of fruits and vegetables. A number of randomized controlled trials have also been conducted in previous years (Table 2), where fruits, vegetables, and spices have proven beneficial for CVD management.

Apple is one of the most commonly consumed fruits, and its polyphenolic extract has a significant effect on decreasing the serum total-C and LDL-C levels in healthy individuals with relatively high body mass index (BMI), which consequently limits CVD risk [32]. Another study showed that the consumption of banana decreased the oxidative modification of LDL, plasma lipids, and lipoproteins and thus ultimately aids in protection from atherogenesis due to its antioxidant properties [33]. In addition, berry fruits (blueberries, strawberries, and cranberries) can also reduce cardiovascular risk factors such as lipid peroxidation, inflammation, and the control of HTN due to the presence of high levels of anthocyanins and ellagitannins in their skin and flesh [34–36]. In addition, for being good sources of polyphenols, berries are rich in micronutrients such as folate, *α*-carotene, *β*-carotene, potassium, vitamin C, and vitamin E, which exhibit noteworthy antioxidant activities [37].

Several studies have shown that citrus fruits such as mandarins, lemons, oranges, and grapefruits contain high quantities of flavanones (e.g., naringin and hesperidin) that improve significant vascular functions and the lipid profile in patients with coronary artery diseases [38–40]. The delicious pomegranate fruit and its juice and peel extracts have antihypertensive, antiatherosclerotic, antioxidant, and anti-inflammatory effects due to the presence of polyphenolic compounds including anthocyanins, catechins, and tannins, which contribute to the attenuation of CVD risk factors [41, 42].

The consumption of polyphenol-rich peach and plum juice showed preventive effects against risk factors for cardiometabolic disorders. This protection was largely achieved by decreasing the expression of plasma proatherogenic and proinflammatory molecules, intercellular cell adhesion molecule-1 (ICAM-1), monocyte chemotactic protein-1, and nuclear factor kappa B (NF-*κ*B) and by decreasing foam cell adherence to aortic arches. In addition, the ingestion of peach and plum juice reduced angiotensin II levels in plasma and reduced the expression of its receptor Agtr1 in cardiac tissues, thus demonstrating the ability of peach and plum polyphenols to act as peroxisome proliferator-activated receptor-*γ* (PPAR*γ*) agonists [43]. An* in vivo* and* ex vivo* experiment by Hong et al. demonstrated that watermelon improved lipid profiles and antioxidant capacity and decreased inflammation, thus altering gene expression for lipid metabolism and consequently reducing the risk factors for CVDs [44].

#### 3.2.2. Vegetables

A group of widely consumed flavonoids present in vegetables exhibit some protective activities against CVD progress [45]. Sulfur-containing organic compounds (organosulfur) from garlic (*Allium sativum*), onion (*Allium cepa*), and cruciferous vegetables such as broccoli, cauliflower, cabbage, and Brussels sprouts showed cardioprotective effects mediated by their antioxidant and anti-inflammatory properties [46]. Moreover, experimental studies on garlic suggested the blocking of platelet aggregation through ADP and platelet-activating factor (PAF) inhibition [47]. A key flavonoid from onion, quercetin (3,3′,4′,5,7-pentahydroxyflavone), exerts antiatherosclerotic effects, and its metabolites, which showed antioxidant and anti-inflammatory activities, accumulate in the aorta tissue [48].

Upaganlawar et al. [69] showed that lycopene, a bright red carotene and carotenoid pigment in tomato, considerably reduced myocardial infarction (MI) in isoproterenol-induced rats because of its antioxidant activities. A recent study (2015) also demonstrated that the supplementation of tomato with corn oil improved diastolic function, changed cardiac miRNA expression, and attenuated both lipid hydroperoxidation and oxidative stress [70]. Similarly, tomato-based products such as tomatoes, tomato sauce, and tomato juice had cardiovascular advantages due to the presence of dietary lycopene [71].

#### 3.2.3. Spices

Several studies have observed that the widely used spice ginger (*Zingiber officinale*) helps in the treatment of CVDs. Ginger exhibits anti-inflammatory as well as antithrombotic properties by inhibiting the production of NO, inflammatory cytokines, cyclooxygenase (COX), and lipoxygenase (LOX), and it shows no or very few side effects, unlike nonsteroidal anti-inflammatory drugs (NSAIDs) [72, 73]. Ginger also displays antioxidant [74], antiplatelet [75], positive inotropic [76], hypotensive [77], and hypoglycemic and hypolipidemic effects [78] in* in vitro* and* in vivo* studies and human clinical trials. One of the most regularly consumed spices, black pepper, and its active principle (piperine) showed significant decreases in the levels of C, free fatty acids, phospholipids, and triglycerides and an increase in the concentration of high density lipoprotein cholesterol (HDL-C), thus reducing the risk of atherosclerosis [79, 80]. Another spice called saffron (*Crocus sativus* L.) and its essential oil-like constituent safranal show remarkable cardioprotective effects in isoproterenol-induced MI Wistar rats by maintaining the redox status of the cell [81, 82]. Cinnamon is another spice that is abundantly found in Bangladesh, India, China, Sri Lanka, Egypt, and Australia; the leaves and barks of cinnamon are used widely in food or to yield essential oils, and they show cardioprotective effects [83].

#### 3.2.4. Miscellaneous

A meta-analysis reported that the consumption of 1 cup/day of green tea could decrease by 10% the chance of developing coronary artery disease due to the presence of polyphenols such as catechins, epicatechin 3-gallate (ECG), and epigallocatechin (EGC) and thereby prevent CVD; however, no significant relationship was found between black tea polyphenols and cardioprotective effects [84].

Chocolate, cocoa, and cocoa products provide a substantial quantity of dietary polyphenols. There is numerous evidence from* in vivo* and* ex vivo* experiments as well as clinical studies showing the roles of these products in protecting against the risk factors of CVDs. A cross-sectional study by Buijsse et al. [85] showed that the consumption of cocoa-containing foods was inversely related to the blood pressure and 15-year cardiovascular mortality. Several meta-analyses have also established that the consumption of cocoa could modulate multiple cardiovascular risk factors such as flow-mediated vascular dilatation, activation of platelets [86], insulin resistance [87], and the blood C level [88].

Because CVD is a multifactorial disorder, the consumption of fruits, vegetables, spices, green tea, red wine, or other polyphenol-rich phytochemicals is expected to decrease the risk of CVD via multiple mechanisms to ensure a healthy life.

## 4. Diabetes Mellitus

DM is a group of metabolic diseases that are caused by overnutrition (mainly high-fat diet) and a lack of physical activity [89]. In healthy individuals, active pancreatic *β*-cells secrete insulin to reduce glucose levels in insulin-sensitive liver, muscle, and adipose tissues [90]. In type 1 diabetes mellitus (T1DM), defective insulin secretion occurs due to dysfunctional pancreatic *β*-cells or a decrease in *β*-cell mass over time, whereas in type 2 diabetes mellitus (T2DM), insulin-stimulated glucose uptake in hepatic and adipose tissues is reduced due to insulin resistance [91]. Over time, insulin resistance tends to increase with age. In contrast, *β*-cells that initially produce insulin in sufficient quantities eventually produce insufficient quantities, thereby leading to the onset and progression of diabetes [92].

Worldwide, DM is the most common endocrine disorder; the reported prevalence in 2013 was 382 million people, which is anticipated to increase to as many as 592 million by 2035 [93]. The majority (80%) of DM patients are from low and middle income countries, where the incidence of DM is expected to increase in the next 22 years [93].

### 4.1. Pathogenesis

Chronic low-grade inflammation and the activation of the innate immune system are considered to be closely involved in the pathogenesis of DM [94]. Excessive levels of nutrients (glucose and free fatty acids) initiate cellular stress in the pancreatic islets and insulin-sensitive tissues including adipose tissue, leading to the activation of c-Jun N-terminal kinase (JNK) and NF-*κ*B (Figure 1) [95]. The inflammatory signaling pathways regulate protein phosphorylation and cellular transcriptional events, thereby increasing the adipocyte production of proinflammatory cytokines, including tumor necrosis factor alpha (TNF-*α*), interleukin (IL) 6, IL-1*β*, leptin, resistin, and chemokines such as MCP-1, CC-chemokine ligand 2 (CCL2), CCL3, and CXC-chemokine ligand 8. As a result, immune cells such as monocytes are recruited to the adipose tissues, thus contributing to tissue inflammation. The monocytes that differentiate into macrophages produce several inflammatory cytokines, further promoting local inflammation. In addition, the release of cytokines and chemokines from the adipose tissues into the circulation promotes inflammation in other tissues including the islets [95].

Both JNK and IKK*β*/NF-*κ*B play important roles in inflammation-induced insulin resistance. JNK is a stress kinase that normally phosphorylates the c-Jun component of the AP-1 transcription factor and promotes insulin resistance through the phosphorylation of serine residues in insulin receptor substrate 1 (IRS-1) [96]. Insulin receptor signaling that normally occurs through a tyrosine kinase cascade [90] is inhibited by counter-regulatory serine/threonine phosphorylation [97]. IKK*β* is highly selective towards its physiological substrates, the I*κ*B protein inhibitors of NF-*κ*B. Phosphorylation by IKK*β* targets I*κ*B*α* for proteasomal degradation, which liberates NF-*κ*B for translocation into the nucleus, where it promotes the expression of numerous target genes whose products induce insulin resistance. IKK*β* causes insulin resistance through the transcriptional activation of NF-*κ*B. Therefore, anti-inflammatory therapies have the potential to decrease gene expression and improve insulin resistance.

Increasing adiposity is reported to increase inflammatory gene expression in the liver [98], which further increases the production of cytokines and chemokines. Immune cells including monocytes and macrophages are recruited and/or activated, which leads to local insulin resistance. Alternatively, the portal delivery of abdominal fat-derived cytokines and lipids contributes to hepatic inflammation. However, NF-*κ*B is activated in hepatocytes, causing the overproduction of cytokines including IL-6, TNF-*α*, and IL-1*β* in fatty liver. The proinflammatory cytokines then contribute to the development of insulin resistance in skeletal muscle and other tissues [95].

Oxidative stress contributes to DM by modifying the enzyme systems, impairing glutathione metabolism and lipid peroxidation and reducing vitamin C levels [99]. In fact, a close relationship exists between hyperglycemia and oxidative stress in DM. Hyperglycemia fuels glucose autooxidation, NADPH oxidase activity, oxidative phosphorylation, protein glycation, and the polyol pathway, which leads to ROS generation and oxidative stress (Figure 1) [100]. ROS attack the healthy cells by damaging the functional and structural integrity of the cells, which consequently leads to many pathophysiological conditions [101].

### 4.2. The Role of Dietary Phytochemicals

Several groups of synthetic drugs and insulin possess antioxidative and anti-inflammatory potential that can be used in the treatment of DM. Unfortunately, none are free from adverse effects (Table 1). Therefore, the quest for alternative and safer treatment regimens for DM is ongoing. In this review, successful clinical trials based on intervention with fruits, vegetables, and spices are considered (Table 2), and* in vivo* and* in vitro* experiments using the phytochemicals from these dietary sources that have exhibited potential to attenuate oxidative stress and inflammation for DM treatment are discussed.

#### 4.2.1. Fruits

Numerous* in vivo* and* in vitro* experiments with fruits, fruit products, and fruit-derived compounds have been extensively conducted for DM management. The oral administration of naringin (4′,5,7-trihydroxyflavonone-7-rhamnoglucoside) at 50 mg/kg/day is reported to reduce oxidative stress and increase fasting plasma insulin in streptozotocin- (STZ-) induced diabetic Sprague Dawley rats. Naringin is considered to be the main flavonoid in grapefruit juice, and it is thought to ameliorate oxidative stress through its antioxidant effects, thereby improving ATP synthesis in pancreatic *β*-cell mitochondria and ameliorating the subsequent insulin secretion by *β*-cells [102]. Another study also investigated the effect of naringin treatment (25, 50, and 100 mg/kg/day) on diabetic Wistar albino male rats for 28 days. Naringin significantly ameliorated *β*-cell dysfunction, insulin resistance, and hyperglycemia, reduced TNF-*α*, IL-6, CRP, and antioxidant enzyme activities and NF-*κ*B expression, and increased adiponectin and PPAR*γ* expression. Additionally, naringin effectively rescued kidney cells, *β*-cells, and liver cells from further pathological modifications and oxidative damage [103].

Resveratrol, a naturally occurring polyphenol found in grapes and red wine, has recently been shown to exert potent antidiabetic, antioxidative, and anti-inflammatory activities. In the liver and spleen of STZ-induced male Long-Evans rats (type 1 diabetic animal models), resveratrol treatment (0.1 or 1.0 mg/kg/day) for 7 days significantly reduced oxidative stress (including superoxide anion content, protein carbonyl level, and manganese-superoxide dismutase expression) in hepatic and splenic tissues, and it reduced hepatic inflammation (NF-*κ*B and IL-1*β*) and decreased the TNF-*α* and IL-6 levels in diabetic spleen [104].

Phlorizin (PZ) is a predominant phenolic compound that is found in apples. Preexposure to docosahexaenoic acid ester of PZ (PZ-DHA) in inflammation-induced macrophages [stimulated by lipopolysaccharide (LPS)] was effective in reducing the TNF-*α*, IL-6, and COX-2 protein levels compared with DHA. Both PZ-DHA ester and DHA have the potential to inhibit NF-*κ*B activation. Therefore, PZ-DHA ester has the potential to be used in T2DM-associated inflammation [105].

Diabetes mellitus is associated with the reduction of glutathione levels, thus indicating the critical role of oxidative stress in its pathogenesis. In Ins-1E pancreatic *β*-cells, pretreatment with the flavonoid epicatechin (present in green tea, grapes, and cocoa) prevented tert-butyl hydroperoxide-induced cell damage, ROS, and p-JNK expression. In addition, it restored insulin secretion which indicates the protective potentiality of epicatechin against oxidative stress on *β*-cells [106].

Pomegranate (*Punica granatum*) fruit contains flavonoids such as flavonols, anthocyanins, ellagitannins, gallotannins, and proanthocyanidins. Pomegranate has been reported to provide a beneficial effect in T2DM by decreasing the lipid peroxidation and oxidative stress by increasing some of the enzymes' antioxidant activity, decreasing the ROS, and preventing or activating PPAR*γ* and NF-*κ*B [107].

Anthocyanins can alter tissue PPAR activity, which further affects metabolism and inflammation. In the Zucker fatty rat model of obesity and metabolic syndrome, the effect of whole tart cherry powder (prepared from anthocyanin-rich tart cherries) was evaluated after 90 days' treatment. The intake of tart cherry reduced retroperitoneal IL-6 and TNF-*α* mRNA expression, NF-*κ*B activity, and plasma IL-6 and TNF-*α* levels, and it increased retroperitoneal PPAR*α* and PPAR*γ* mRNA expression. As a whole, tart cherry consumption reduced both systemic and local inflammation and metabolic syndrome, which may reduce the risk of T2DM development [108].

Macrophage infiltration in adipose tissue due to increased adiposity can lead to T2DM. In an* in vitro* model of inflammation in which the pathologic relationships between adipocytes and macrophages were mimicked, anthocyanin-enriched fractions from blackberry-blueberry beverages were found to inhibit the secretion of NO and TNF-*α* and the phosphorylation of NF-*κ*B p65 in LPS-induced macrophages [109].

T2DM is associated with chronic, low-grade, systemic inflammation accompanied by an increased production of adipokines or cytokines by obese adipose tissue. The treatment of diabetic db/db mice with grapefruit (0.5 g/kg) for six weeks produced antihyperglycemic effects that were accompanied by the reduced mRNA expression of proinflammatory genes such as COX-2, monocyte chemotactic protein-1, TNF-*α*, and NF-*κ*B in the liver and epididymal adipose tissue. Hypermethylation at the CpG3 site of TNF*α* in adipose tissue was also found, which may contribute to a reduction of the inflammation associated with T2DM [110].

In pancreas, liver, and adipose tissue, endoplasmic reticulum (ER) stress is an early event linked to T2DM pathogenesis. In the skeletal muscle of diabetic rats, 500 mg/kg of grape seed proanthocyanidin extract (GSPE) administration for 16 weeks decreased the plasma glucose levels and insulin resistance, restored the normal activities of antioxidant enzymes and ATPases, and partially alleviated severe ER stress, which suggests that GSPE could be a useful treatment strategy for T2DM [111]. A similar study on GSPE using human adipocytes (SGBS) and macrophage-like (THP-1) cells found reduced cytokine (IL-6 and MCP-1) gene expression after an inflammatory stimulus and enhanced the production of the anti-inflammatory adipokines adiponectin (APM1) and LEP, which may prevent the low-grade inflammation of T2DM [112].

Milk fat globule epidermal growth factor-8 (MFG-E8) is highly involved in the inflammatory response. In diabetic db/db mice, the administration of grape seed procyanidin B2 (a natural complex of polyphenol polymers) provided anti-inflammatory protection in pancreatic tissues by downregulating MFG-E8 and attenuating the levels of the proinflammatory cytokines IL-1*β* and NLRP3 [113]. Because the dysfunction of pancreatic islets is one of the mainstays in T2DM pathogenesis, protecting the pancreas from inflammation may lead to potential therapeutic approaches.

#### 4.2.2. Vegetables

There is abundant* in vitro* and* in vivo* evidence that vegetables have anti-inflammatory and antioxidant potential for DM management. In diabetic Wistar rats, the immunomodulatory effects of a mycelial submerged culture and the broth of* Grifola frondosa* mushrooms were explored on splenocytes and peripheral blood cells. Two weeks of intragastric administration of fermented mycelia, broth, or their combination (1 g/kg/day) significantly decreased the 2-hour postprandial blood glucose level, the production of T-leukocyte-derived interferon gamma (IFN-*γ*), monocyte-derived IL-4 and IL-6, and T-splenocyte-derived IL-4, and this treatment significantly enhanced macrophage-derived TNF-*α* production [114].

In STZ-induced diabetic rats, the administration of fermented carrot juice by* Lactobacillus plantarum* NCU116 for five weeks positively regulated the blood glucose level, hormone, and lipid metabolism, reestablished the antioxidant capacity, restored the morphology of pancreas and kidney, and upregulated the LDL receptor, cholesterol 7*α*-hydroxylase (CYP7A1), GLUT4, and PPAR*α* and PPAR*γ* mRNA expression [115].

Sulforaphane (SFN) is an isothiocyanate that is naturally available in widely consumed vegetables, particularly broccoli. In diabetic male C57BL/6J mice, sulforaphane (0.5 mg/kg) treatment for four months significantly inhibited cardiac lipid accumulation and improved cardiac inflammation, oxidative stress, and fibrosis by downregulating diabetes-induced PAI-1, TNF-*α*, CTGF, TGF-*β*, 3-NT, and 4-HNE expression. SFN also upregulated nuclear factor (erythroid-derived 2-) like factor 2 (Nrf2) and its downstream genes, NQO1 and HO-1; SFN decreased 4-HNE-LKB1 adducts and reversed the diabetes-induced inhibition of LKB1/AMPK and its downstream targets, including sirtuin 1, PGC-1*α*, phosphorylated acetyl-CoA carboxylase, and carnitine palmitoyl transferase-1. These results suggest that the SFN treatment of T2DM mice may attenuate the cardiac oxidative stress-induced inhibition of the LKB1/AMPK signaling pathway, thereby preventing T2DM-induced lipotoxicity and cardiomyopathy [116].

Onion-derived quercetin derivatives have been regarded as the most important flavonoids for improving diabetic conditions in both* in vivo* and* in vitro* models. In STZ-induced male Sprague Dawley rats, eight days of treatment with onion peel extract (1%) significantly (*p* = 0.0148) improved glucose tolerance, liver and skeletal muscle glycogen content (*p* < 0.0001 and *p* = 0.0089, resp.), and insulin receptor (*p* = 0.0408) and GLUT4 (*p* = 0.0346) expression in muscle tissues. The oxidative stress-inducing activities, such as superoxide dismutase activity, the formation of malondialdehyde, free fatty acids in the plasma, and IL-6 expression in hepatic protein, were significantly (*p* = 0.0393, 0.0237, 0.0148, and 0.0025, resp.) decreased [117].

Cordycepin (3′-deoxyadenosine) is produced by a traditional medicinal mushroom known as* Cordyceps militaris*. Although cordycepin has been shown to exert immunological stimulation and anti-infection and anticancer activities, the molecular mechanisms of cordycepin in T2DM are unclear. In LPS-stimulated RAW 264.7 cells, cordycepin has been found to inhibit NO, suppress NF-*κ*B activation, and suppress the protein expression of proinflammatory mediators that further inhibit the production of proinflammatory cytokines such as IL-1*β*, IL-6, and TNF-*α*. Moreover, an increased concentration of cordycepin decreased the T2DM-regulating genes such as 11*β*-HSD1 and PPAR*γ* as well as the expression of costimulatory molecules such as ICAM-1 and B7-1/-2 [118].

The administration of MT-*α*-glucan (isolated from* Grifola frondosa* mushroom fruit body), in a murine T2DM model, significantly decreased the body weight, fasting plasma glucose levels, HbA1c, TG, cholesterol, FAA, NO, NO synthase, inducible NO synthase, and malondialdehyde content in the liver. MT-*α*-glucan also significantly increased the serum insulin content and hepatic glycogen content, and it reduced both the glutathione levels and the superoxide dismutase and glutathione peroxidase activity. These results suggest that the hypoglycemic effects of MT-*α*-glucan in T2DM mice might be connected to its protection of pancreatic *β*-cells accomplished by decreasing oxidative stress and NO synthesis [119].

In an* in vitro* study, cocoa (*Theobroma cacao*) extract has shown dose-dependent inhibition of *α*-amylase, *α*-glucosidase, and angiotensin-1-converting enzyme activities and has also shown scavenging ability for several radicals [DPPH (16.94 ± 1.34 mg/mL), NO (6.98 ± 0.886 mg/mL), OH (3.72 ± 0.26 mg/mL), and ABTS (15.7 ± 1.06 mmol/TEAC·g)] [120].

#### 4.2.3. Spices

A variety of spices also show potential for managing DM by reducing inflammation and oxidative stress. Turmeric (*Curcuma longa*) contains curcumin (a polyphenolic compound) as the active ingredient, which possesses broad-spectrum biological actions such as anti-inflammatory, antioxidant, and antitumor activities. In the injured lungs of diabetic rats, curcumin has been found to reduce oxidative stress and inflammatory responses and inhibit prostaglandin E2 (PGE2) and NOS. Further results revealed that curcumin inhibited the activation of NF-*κ*B, which is a key player in inflammatory responses [121]. In db/db mice, curcumin treatment for eight weeks increased AMPK and PPAR*γ* expression and diminished NF-*κ*B protein levels [122]. In T1DM patients, the development of skeletal muscle atrophy is associated with chronic inflammation. According to an* in vivo* study on STZ-induced T1DM C57BL/6J mice, curcumin at a dose of 1500 mg/kg/day for two weeks ameliorated skeletal muscle atrophy by inhibiting NF-*κ*B activation, inflammatory cytokine (TNF-*α* and IL-1*β*) concentrations, oxidative stress, and protein ubiquitination [123]. According to an* in vitro* study, the treatment of solid lipid curcumin particle (SLCP) formulations (10 to 50 *μ*g/mL) using LPS-stimulated RAW 264.7 cultured murine macrophages significantly decreased the LPS-induced proinflammatory mediators NO, PGE2, and IL-6 by inhibiting the activation of NF-*κ*B [124]. In another study on STZ-induced diabetic Wistar-NIN rats, treatment with 0.01% curcumin or 0.5% turmeric for a period of eight weeks controlled the oxidative stress and restored normal antioxidant enzyme activities [125]. Neuronal injury can be induced by hyperglycemia-mediated oxidative stress due to diabetes. Curcuminoids, which are polyphenols of turmeric, exhibited protective effects against oxidative stress in the brain of STZ-induced diabetic rats by restoring the normal level of lipid peroxidation and nitrite content and endogenous antioxidant marker enzymes [126]. In an* in vitro* study, curcumin was found to attenuate insulin-induced oxidative stress in hepatic stellate cells by inducing the expression of glutamate-cysteine ligase, leading to the de novo synthesis of glutathione and the suppression of insulin receptor expression [127]. In an* in vitro* study, pretreatment with a novel curcumin analogue (B06) at 5 *μ*M significantly reduced the high glucose-induced overexpression of inflammatory cytokines in macrophages via the inhibition of c-Jun N-terminal kinase/NF-*κ*B activation [128].

Diabetic complications occur as a result of increased ROS due to long-term hyperglycemia. Honey and ginger have been shown to exhibit antioxidant activity by scavenging ROS. In STZ-induced Sprague Dawley rats, the combined administration of honey (2 g/kg body weight) and ginger (60 mg/kg body weight) for three weeks significantly (*p* < 0.05) reduced the superoxide dismutase and catalase activities and the malondialdehyde levels, whereas the reduced glutathione level and the reduced glutathione/oxidized glutathione ratio were significantly elevated (*p* < 0.05) [129]. In STZ-induced diabetic rats, oral administration with the combined extract of purple waxy corn and ginger at doses of 100, 200, and 300 mg/kg body weight for 21 days improved chronic constriction at the right sciatic nerve by improving the oxidative stress status and the axon density in the lesion nerve [130]. Ginger was also found to inhibit *α*-glucosidase and *α*-amylase enzymes, which is useful for T2DM management, and inhibition of COX was observed, which protects against inflammation [131]. The oral administration of ginger in diabetic rats was found to exert neuroprotective effects by increasing antioxidant defense mechanisms and downregulating malondialdehyde (MDA) levels to the normal levels in brain [132]. In another similar study, ginger administered at a dose of 500 mg/kg/day revealed a protective role on diabetic brain accomplished by reducing oxidative stress, apoptosis, and inflammation in STZ-induced diabetic rats [133]. According to another* in vivo* study, the treatment of STZ-induced inbred male Wistar/NIN rats for one month with ginger powder (0.5%, 1%, and 5%) showed protective effects against diabetes by modulating antioxidant enzymes and glutathione and downregulating lipid and protein oxidation [134]. In nicotinamide and STZ-induced diabetic rats, treatment with garlic bulb, ginger rhizome, turmeric rhizome, and their mixture (200 mg/kg body weight) for 28 consecutive days significantly alleviated hyperglycemia and dyslipidemia, increased insulin production, enhanced GSH, and decreased lipid peroxidation [135].

In diabetic patients, diabetic encephalopathy is one of the more severe complications. In diabetic encephalopathy rats, saffron at 40 and 80 mg/kg significantly increased the body weight and serum TNF-*α* levels and decreased the blood glucose, glycosylated proteins, and advanced glycation end product (AGE) levels in serum. Furthermore, saffron significantly increased the glutathione content, superoxide dismutase, and catalase but remarkably decreased the cognitive deficit and serum TNF-*α*, and it induced NOS activity in hippocampus tissue [136]. In STZ-induced diabetic rats with renal injury, the administration of crocin, an active constituent of saffron, significantly decreased malondialdehyde (*p* < 0.01) and xanthine oxidase (*p* < 0.05) activities and elevated glutathione (*p* < 0.05) levels, thus ameliorating renal injury [137]. Safranal is one of the components of the saffron plant. In high-fat diet (HFD) and STZ-induced T2DM rats, safranal treatment for a period of four weeks decreased the oxidative stress caused by T2DM and reduced the inflammation by reducing the TNF-*α* and IL-1*β* levels in the plasma and pancreas tissue [138].

The protective effect of onion against oxidative stress was evaluated in an* in vivo* study. In STZ-induced male diabetic Wistar rats, daily treatment with 1 mL of* Allium cepa* solution (0.4 g* Allium cepa*/rat) increased the fasting serum high density lipoprotein levels and alleviated hyperglycemia by decreasing superoxide dismutase activities [139]. Another* in vivo* study also investigated the protective effects of onion against oxidative stress; 12 weeks of onion intake suppressed the diabetes-induced oxidative stress more effectively in STZ-induced diabetic rats [140]. In STZ-induced diabetic male Sprague Dawley rats, supplementation with onion powder (7% w/w) suppressed the glutathione peroxidase, glutathione reductase, and glutathione S-transferase activities from high to normal levels [141].

Mustard leaf (*Brassica juncea*) has been reported to strongly inhibit the formation of AGE and free radical-mediated protein damage in* in vitro* studies. According to an* in vivo* study with STZ-induced diabetic rats, the oral administration of the EtOAc fraction of mustard leaf at doses of 50 and 200 mg/kg body weight/day for 10 days reduced the serum levels of glucose and glycosylated protein as well as the superoxide and nitrite/nitrate levels, which suggests that the EtOAc fraction of mustard leaf has the capacity to attenuate damage caused by the oxidative stress involved in diabetes and its complications [142].

Sesame butter is a natural product produced by grinding sesame seeds and is thus free from any chemical or nonchemical additives. In STZ-induced male albino Wistar rats, oral treatment with sesame butter (1.25 g/kg) for six weeks significantly decreased the blood glucose, high density lipoprotein, and malondialdehyde levels and increased the total antioxidant capacity [143]. Sesame contains sesamin, a lignan that has been found to diminish the elevation of malondialdehyde and the reduction of superoxide dismutase activity induced by diabetes after seven weeks of treatment in male diabetic rats [144].

Eugenol (EU) is an active principle of cloves (*Syzygium aromaticum*) that can also be found in basil and cinnamon. In an* in vitro* study with SHSY5Y cells under experimentally induced hyperglycemic conditions, the exposure of cells to EU (5–10 *μ*M) improved cell viability, reduced the glutathione (GSH) levels, and significantly decreased the glucose-associated oxidative stress (by diminishing ROS and peroxide levels). From the* in vivo* experiment on STZ-induced diabetic rats, treatment with EU at a dose of 10 mg/kg body weight/day for six weeks diminished the oxidative marker levels, GSH, and total thiols and enhanced the antioxidants activities [145]. In high-fat-diet and STZ-induced T2DM rats, the administration of clove bud powder (20–40 g/kg) reduced the blood glucose level and *α*-glucosidase and liver enzyme (alanine aminotransferase, aspartate aminotransferase, and alkaline phosphatase) activities and elevated the antioxidant (glutathione, ascorbic acid, superoxide dismutase, and catalase) levels [146].

Cumin (*Cuminum cyminum*) is widely used as a spice in many countries. In STZ-induced diabetic rats, treatment with a methanolic extract of* C. cyminum* seeds for 28 days effectively controlled the oxidative stress, inhibited AGE formation, and improved the antioxidant status in the kidney and pancreas [147].

## 5. Rheumatoid Arthritis

RA is an inflammatory, systemic autoimmune disorder with primary degenerating articular structures involving, in particular, the cartilage (movable synovial joints of knees, shoulders, and hands) and the bones (osteoarthritis and osteoporosis) as a result of pannus formation over the joint surfaces (abnormal layer of fibrovascular or granulation tissues) [148, 149]. The symptoms include swelling, warmth, and redness of the joints with pain, morning stiffness, fatigue, and limited functioning of the joints, which can result in poor coordination of the limbs and the deterioration of the posture [150, 151].

RA is the most common inflammatory arthritis and affects approximately 1% of the world population, with more than 3 million new patients being diagnosed yearly [152, 153]. It has been reported that 80% of RA patients become disabled within 20 years of diagnosis, and if left untreated, approximately 20–30% of RA patients can be permanently disabled within 2-3 years of their first diagnosis [154, 155]. In addition, women are three times more likely to be affected than men [156]. According to a 2013 report, one in every five adults (22.7%) in the United States has been diagnosed with arthritis [157], and the number of affected individuals is projected to increase to approximately 67 million by 2030 [158]; this is going to be an alarming health issue imposing a great socioeconomic impact worldwide.

### 5.1. Pathogenesis

The pathogenesis of RA involves a complex interplay between genetic and environmental factors leading to autoimmune inflammatory responses against the connective and synovial tissues of the joints [3]. In addition, increased ROS levels are actively involved in RA pathogenesis (Figure 1) [159, 160]. In typical physiological conditions, different types of cytokines are actively present in synovial tissues. However, in patients with RA, immune cells including T cells, B cells, and macrophages penetrate the affected synovial tissues and promote the overexpression, release, and activity of proinflammatory cytokines including TNF-*α*, TNF-induced NF-*κ*B, vascular endothelial growth factor (VEGF), IL-1 beta (IL-1*β*), IL-6, IL-8, and IFN-*γ* [3, 161, 162]. In response to these proinflammatory cytokines, fibroblast-like synoviocytes (FLS) proliferate and produce large quantities of cytokines, matrix metalloproteinases (MMPs), and COX-2, which progressively degrade cartilage and lead to joint destruction (Figure 1) [163, 164].

Oxidative stress was recently shown to be involved in the degeneration of cartilage due to the deregulation of Nrf2 or NFE2L2 [165]. When Nrf2 is activated, it binds to antioxidant response elements (AREs), resulting in the increased expression of antioxidative enzyme [e.g., Heme oxygenase-1 (HO-1)] encoding genes [166, 167]. Thus, both oxidative stress and inflammatory processes are implicated in the pathogenesis of RA.

### 5.2. The Role of Dietary Phytochemicals

Although synthetic regimens have been used to treat patients with RA, side effects are common and unavoidable (Table 1). Researchers have found that the regular consumption of fresh fruits, vegetables, and spices rich in important phytochemicals can attenuate oxidative stress and inflammation and relieve RA. According to some cohort studies on RA, high consumption of fruits and vegetables not only is inversely correlated with disease progression but also exerts some protective effects against RA [168–170]. Here, we discuss some of the clinical (Table 2) and experimental evidence of phytochemicals being successfully used as alternative treatments against RA.

#### 5.2.1. Fruits and Vegetables

Osteoclastogenesis (the process of destroying bone tissues by osteoclast cells) has been observed as a clinical phenomenon in patients with RA [171]. Polyphenols extracted from dried plums can inhibit osteoclastogenesis by suppressing the activity of TNF-*α* and NO synthase and downregulating the transcription factor-nuclear factor for activated T cells (NFATc1) [172]. Therefore, polyphenols have the potential to be used in RA treatment.

Anthocyanins from cherry can reduce both oxidative stress (increase superoxide dismutase and decrease serum malondialdehyde) and inflammatory mediators (decrease in TNF-*α* levels) in an adjuvant-induced RA rat model (male Sprague Dawley) [173].

In 2015, resveratrol polyphenol found in red grape skin was reported to confer a significant protective effect against an aggressive RA rat model [174]. In the experiment, the activity of specific rheumatoid biomarkers [serum rheumatoid factor (RF), MMP-3 and cartilage oligomeric matrix protein (COMP)], immunological biomarkers [IgG and antinuclear antibody (ANA)], immunomodulatory cytokines (TNF-*α*), and oxidative stress biomarkers [myeloperoxidase (MPO), CRP, and MDA] were significantly reduced by the anti-inflammatory and antioxidative activities of resveratrol.

In an* in vivo* study with RA-induced DBA-1/J male mice, mangiferin (a natural polyphenol found especially in mangoes) suppressed the expression of IL-1*β*, IL-6, TNF-*α*, and receptor activator of NF-*κ*B ligand (RANKL) via the activation of extracellular signal-regulated kinase 1/2 (ERK1/2) and the inhibition of NF-*κ*B [175]. Another study with mangiferin demonstrated protection against joint destruction in RA by exerting strong proapoptotic effects on human synovia-derived synoviocytes [176].

Recently, Natarajan et al. [177] showed the suitability of using intra-articular injections with polyphenols to protect against cartilage degradation in collagen-induced arthritic rats. They injected a combination of different polyphenols (EGC, gallate, catechin, tannic acid, and quercetin) in RA-induced rats and observed significant (*p* < 0.05) protection effect against cartilage degradation along with attenuated inflammation.

Kaempferol (found especially in grapefruits) was shown to inhibit synovial fibroblast proliferation by suppressing inflammatory cytokines (inhibiting IL-1*β*), inhibiting the phosphorylation of ERK-1/2, p38, and JNK, inhibiting the activation of NF-*κ*B, and reducing oxidative stress by inhibiting the production of MMPs, COX-2, and PGE2 in RA-derived synovial fibroblasts [178]. All of these cytokines, transcription factors, cell signaling pathways, and enzymes are established compounds in the pathogenesis of RA that in combination destroy the articular bone and cartilage in patients with RA [179, 180].

An* in vivo* study on* p*-coumaric acid (a polyphenol present in grapes, apples, oranges, spinach, tomatoes, potatoes, wheat, oats, and maize) confirmed its potent immunosuppressive activity because it significantly (*p* < 0.05) reduced the expression of TNF-*α* in adjuvant-induced arthritic rats [181]. In a collagen-induced RA rat model (female Sprague Dawley), genistein (a polyphenol rich in soybeans) exerted anti-inflammatory activities; it maintained a balance between the T helper cell-1 (Th1) and Th2 cells by significantly suppressing IFN-*γ* and augmenting the production of IL-4 [182].

Pattison et al. [183] reported that the modest intake of beta-cryptoxanthin (a natural carotenoid) via the daily consumption of a glass of freshly squeezed orange juice is inversely correlated with the risk of RA in humans. Additionally, arterial dysfunction is a common clinical manifestation in patients with RA that leads to cardiovascular complications [184, 185]. Nevertheless, daily vegetable consumption was significantly (*p* = 0.002) associated with more favorable arterial function in patients with RA [186].

#### 5.2.2. Spices

An* in vivo* study [187] revealed the beneficial effects of using a mixture of blended ginger, which is rich in pungent phenolic compounds (e.g*.,* shogaols and gingerols), and turmeric, which is rich in phenolic curcuminoids (including curcumin, bisdemethoxycurcumin, and demethoxycurcumin), against extra-articular complications of RA including hematological, metabolic, and cardiovascular complications in adjuvant-induced arthritic rats (male Wistar albino). Earlier, the same group of researchers confirmed that both ginger and turmeric could independently and significantly (*p* < 0.05) protect against RA in adjuvant-induced arthritic male Wistar albino rats [188].

Cinnamon bark (*Cinnamomum zeylanicum*), one of the most common spices used in Indian, Bangladeshi, Burmese, and Sri Lankan dishes, can confer some protective effects against RA. Rathi et al. [189] observed a significantly (*p* < 0.001) higher level of anti-inflammatory activities [inhibition of cytokines (IL-2, IL-4, and IFN-*γ*) and reduction of TNF-*α* concentration] upon the treatment of RA animal models (male Wistar rat and Swiss albino mice) with the polyphenolic fractions of cinnamon bark.

An* in vitro* study with FLS (derived from RA patients) demonstrated that curcumin is a potent anti-inflammatory spice [190] that blocks the expression of IL-1*β* and IL-6, which are believed to play crucial roles in RA pathogenesis [191]. Elevated apoptosis within the joint is therapeutically useful [192], and curcumin has been found to increase the levels of apoptosis in rheumatic FLS [190].

Methotrexate is an antirheumatic drug widely used to treat patients with RA [193]. Vascular endothelial dysfunction has been reported as one of its most deleterious side effects due to its ability to increase oxidative stress and decrease NO levels [194]. The coadministration of curcumin with folic acid was found to abrogate methotrexate-induced vascular endothelial dysfunctions in male Wistar rats due to the attenuation of oxidative stress and the regulation of NO production [195].

Another recent* in vivo* study on male Sprague Dawley rats [196] revealed that the administration of curcumin could decrease the expression of NF-*κ*B, TNF-*α*, and IL-1*β* in both synovial fluid and blood serum, thus producing effects similar to a standard antirheumatic regimen with methotrexate. Therefore, it is anticipated that curcumin could be given as antirheumatic therapy for patients with RA either administered alone or as an adjuvant with modern therapies.

#### 5.2.3. Miscellaneous

Extra virgin olive oil (EVOO) is consumed all over the world, especially in the Mediterranean countries [197]. In 2014 [198], a group of Spanish researchers observed that polyphenolic extracts of EVOO are potent anti-inflammatory substances protecting against RA-associated inflammation. An* in vivo* experiment conducted with a group (*n* = 10) of collagen-induced RA mice (male DBA-1/J) confirmed that polyphenolic extracts of EVOO inhibit JNK (associated with the regulation of cytokine function, T-cell differentiation, and apoptosis), p38 [involved in mitogen-activated protein kinase (MAPK) pathway, regulating cytokines and apoptosis], and signal transducer and activator of transcription-3 (STAT-3) and decrease the translocation of NF-*κ*B. Interestingly, the activation of JNK, p38, and NF-*κ*B and the overexpression of STAT-3 actively contribute to the pathogenesis of RA [180, 199, 200]. Therefore, EVOO is a potential natural source of polyphenols that can combat RA.

A recent experiment in 2015 [201] with a green tea-derived polyphenolic compound called epigallocatechin-3-gallate (EGCG) demonstrated that EGCG significantly (*p* < 0.01) upregulates the expression of the Nrf-2 antioxidant pathway in mice (male DBA-1/J) with RA. Yoon et al. [202] observed in FLS that gallic acid (a polyphenol found in grapes, tea, gall nuts, sumac, and wine) reduced the expression levels of proinflammatory cytokines (IL-1*β* and IL-6) and enzymes, including MMP-9 and COX-2, which are involved in the inflammation and oxidation-induced pathogenesis of RA. However, the black tea-derived polyphenol theaflavin-3,3′-digallate (TFDG) demonstrated some protective activity against osteoclast formation and osteoporosis via the inhibition of MMP-2 and MMP-9, both of which are responsible for the degradation of collagens and joint destruction in RA patients [203, 204].

An* in vivo* study with cocoa-extracted polyphenols reported the suppression of TNF-*α*-induced VEGF expression (involved in RA) via the inhibition of phosphoinositide 3-kinase (PI3 K) and MAPK kinase-1 (MAP2K1) activities. Grape polyphenolic extracts have been confirmed to have potent anti-inflammatory activity against RA-mediated inflammation by attenuating the expression of TNF-*α*, IL1*β*, IL-6, and IFN-*γ*, and they can reduce the “arthritis score” in experimental rats [205].

In LPS-stimulated macrophages, the secretion of IL-6 (found at elevated levels in patients with RA) was significantly reduced (by at least 25%) upon incubation with polyphenol-rich extracts of rooibos tea, black pepper, ginger, allspice, caraway, bay leaves, cinnamon, licorice, paprika, clove, nutmeg, and apples [206]. In addition, decreased TNF-*α* secretion (by at least 25%) was observed upon incubation with chili pepper, black pepper, cinnamon, bay leaf, caraway, licorice, nutmeg, and bilberry extracts containing high concentrations of polyphenols [206].

## 6. Conclusion

Dietary phytochemicals are some of the most potential natural sources for developing novel drugs with improved efficiency, efficacy, and safety. Well-designed clinical trials are warranted to address the safety issues and the concurrent utility of synthetic drugs and natural compounds in attenuating oxidative stress and inflammation-mediated degenerative diseases such as RA, DM, and CVD. In* in vitro* and* in vivo* or* ex vivo* experiments, more emphasis should be given to studies with active compounds extracted from natural mixtures of phytochemicals. In addition, it would be interesting to conduct computational* in silico* analyses to determine compatible phytochemicals targeting the active sites of regulatory proteins associated with CVDs, DM, and RA to promote the development of safer and more effective drugs.

## Figures and Tables

**Table 1:** Mechanism of action and side effects of some synthetic regimens used in the treatment of CVD, DM, and RA.

Number	Drugs (brand names)	Mechanism	Side effects	Structure
*Cardiovascular diseases*				

1	Aspirin (Easprin®)	(i) Inhibiting the production of TXA_2_ by inactivation of COX-1 and COX-2 enzymes	(i) Stomach bleeding (ii) Risk of hemorrhagic stroke ↑ (iii) Gastric ulcers (iv) Stomach upset (v) Heartburn (vi) Nausea (vii) Epigastric distress (viii) Dyspepsia (ix) Thrombocytopenia (x) Fibrinolytic activity ↑	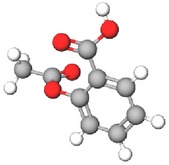

2	Quinapril (Accupril®)	(i) Inhibiting ACE which catalyzes the formation of angiotensin II (a strong vasoconstrictor)	(i) Dizziness (ii) Lightheadedness (iii) Tiredness (iv) Dry cough (v) Nausea (vi) Vomiting (vii) Blurred vision (viii) Chest pain (ix) Confusion (x) Sweating	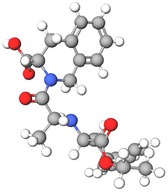

3	Digoxin (Lanoxin®)	(i) Inhibiting the Na-K-ATPase membrane pump resulting in an increase in intracellular sodium	(i) Dizziness (ii) Fainting (iii) Fast pounding or irregular heartbeat or pulse (iv) Slow heartbeat (v) Nausea (vi) Vomiting (vii) Headache (viii) Loss of appetite (ix) Diarrhea	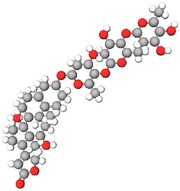

4	Amlodipine (Norvasc®)	(i) Decreasing arterial smooth muscle contractility and vasoconstriction by inhibiting the influx of calcium ions through calcium channels	(i) Dizziness (ii) Lightheadedness (iii) Swelling ankles/feet (iv) Flushing (v) Fatigue (vi) Palpitations (vii) Edema (viii) Tightness in the chest (ix) Wheezing	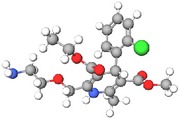

5	Isosorbide dinitrate (Dilatrate-SR®)	(i) It is converted into NO which activates the enzyme guanylate cyclase that stimulates the synthesis of cGMP which then activates a series of protein kinase-dependent phosphorylations in the smooth muscle cells eventually resulting in vasodilation	(i) Headache (ii) Dizziness (iii) Lightheadedness (iv) Nausea (v) Flushing (vi) Unusual bleeding (vii) Bruising (viii) Rapid heart rate (ix) Difficulty with breathing	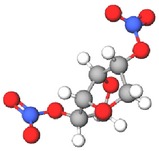

*Diabetes mellitus*				

1	Metformin (Glucophage®)	(i) Activating AMPK to inhibit hepatic glucose production (ii) Increasing AMPK activity in skeletal muscle for glucose uptake by GLUT4 deployment to the plasma membrane	(i) Lactic acidosis (ii) Dyspepsia (iii) Nausea (iv) Diarrhea (v) Vomiting (vi) Weakness	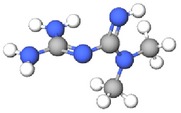

2	Pioglitazone (Actos®)	(i) Activating PPAR*γ* receptors to modulate the insulin-sensitive genes transcription for the reduction of insulin resistance	(i) Weight gain (ii) Risk of bladder cancer (iii) Sore throat (iv) Muscle pain (v) Tooth problems (vi) May cause heart failure (vii) Fluid retention (viii) Peripheral edema	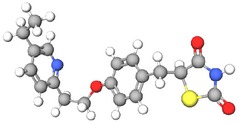

3	Glibenclamide (DiaBeta®)	(i) Binding and inhibiting the ATP-sensitive potassium channels SUR1 receptor on the pancreatic cell surface, which causes membrane depolarization and opens the calcium channels for insulin exocytosis	(i) Nausea (ii) Vomiting (iii) Diarrhea (iv) Increased appetite (v) Heartburn (vi) Stomach fullness (vii) Weight gain (viii) Hypoglycemia	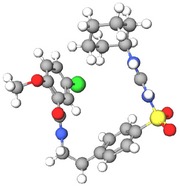

4	Acarbose (Precose®)	(i) Reversibly binding to pancreatic *α*-amylase and intestinal *α*-glucoside hydrolases to inhibit the hydrolysis of complex carbohydrates to glucose	(i) Diarrhea (ii) Gas (iii) Upset stomach (iv) Constipation (v) Stomach pain (vi) Nausea (vii) Loss of appetite (viii) Jaundice	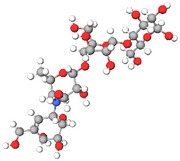

5	Human insulin (Humulin R®)	(i) Binding to the IR and stimulating the downstream signaling molecules which regulates the GLU4 and PKC activity	(i) Pain (ii) Mild itching (iii) Redness (iv) Swelling at the injection site (v) Mild weight gain (vi) Hypoglycemia	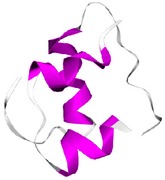

*Rheumatoid arthritis*				

1	Methotrexate (Trexall®)	(i) Inhibiting dihydrofolate reductase which is involved in purine metabolism (ii) Inhibiting T-cell activation (iii) Selectively downregulating B cells (iv) Inhibiting methyltransferase activity, which leads to deactivation of immune system enzyme activities	(i) Nausea (ii) Bloody vomiting (iii) Stomach pain (iv) Drowsiness (v) Dizziness (vi) Black, tarry stools (vii) Sores in the mouth or lips (viii) Blurred vision (ix) Confusion (x) Convulsions	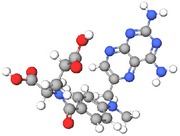

2	Hydroxychloroquine (Quineprox®)	(i) Inhibiting stimulation of the TLR-9 (ii) Inhibiting the production of rheumatoid factor	(i) Nausea (ii) Stomach cramps (iii) Loss of appetite (iv) Diarrhea (v) Dizziness (vi) Headache (vii) Convulsions (viii) Weakness (ix) Sore throat (x) Fever (xi) Unusual bleeding (xii) Bruising	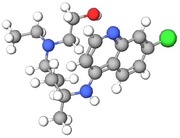

3	Sulfasalazine (Azulfidine®)	(i) Inhibiting NF-*κ*B and exerting anti-inflammatory and/or immunomodulatory activities	(i) Stomach upset (ii) Nausea (iii) Vomiting (iv) Loss of appetite (v) Headache (vi) Dizziness (vii) Unusual tiredness (viii) Fever (ix) Sore throat (x) Skin rash or itching (xi) Bleeding gums (xii) Dark urine	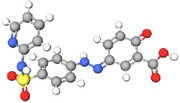

4	Leflunomide (Arava®)	(i) Inhibiting mitochondrial enzyme dihydroorotate dehydrogenase and thus inhibiting the reproduction of rapidly dividing cells, especially autoimmune lymphocytes	(i) Diarrhea (ii) Nausea (iii) Dizziness (iv) Skin rash (v) Alopecia (vi) AST ↑ (vii) ALT ↑ (viii) Bloody or cloudy urine (ix) Cough (x) Headache (xi) Vomiting (xii) Sore throat	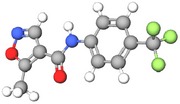

5	Certolizumab (Cimzia®)	(i) Binding and neutralizing the activity of TNF*α* (ii) Inhibiting the release of IL-1*β* from monocytes	(i) Fever (ii) Cough (iii) Sore throat (iv) Runny nose (v) Frequent urination with burning sensation (vi) Joint pain (vii) Body aches (viii) Headache (ix) Loss of voice (x) Ear congestion	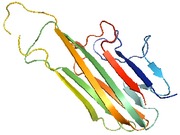

AMPK: AMP-activated protein kinase; GLUT4: glucose transporter 4; SUR1: sulfonylurea receptor 1; IR: insulin receptor, PKC: protein kinase C; PPAR*γ*: peroxisome proliferator-activated receptor gamma; ATP: adenosine triphosphate; TXA2: thromboxane A2; COX: cyclooxygenase: AST: aspartate aminotransferase; ALT: alanine aminotransferase; ACE: angiotensin converting enzyme; NO: nitric oxide; cGMP: cyclic guanosine 3′,5′-monophosphate.

**Table 2:** Evidence of clinical trials with natural products attenuating oxidative stress and inflammation in patients with CVD, DM, and RA.

Number	Type of study	Number of participants	Age (years)	Intervention	Comparison/control	Period of intervention	Outcomes	Year [references]
*Cardiovascular disease (CVD)*								

1	Randomized controlled crossover trial	30 (F = 24, M = 6)	47.3 ± 13.6	Control, apple, spinach, and apple + spinach	Healthy control	4 weeks	All treatments showed higher flow-mediated dilatation (FMD) (*p* < 0.05) and lower pulse pressure (*p* < 0.05) and apple and spinach resulted in lower systolic blood pressure (*p* < 0.05)	2012 [49]

2	Double-blind randomized crossover trial	M = 30	52.6 ± 5.5	Two lyophilized apples (40 g), polyphenol-rich and polyphenol-poor, providing, respectively, 1.43 g and 0.21 g polyphenols per day	Hypercholesterolemic	6 weeks	FMD did not differ between the polyphenol-rich and the polyphenol-poor apples, neither did the other cardiovascular disease risk factors (plasma lipids, homocysteine, and antioxidant capacity)	2010 [50]

3	Open prospective randomized crossover controlled feeding trial	40 (F = 21, M = 19)	28 ± 11	Raw tomatoes/kg (7.0 g), 3.5 g of tomato sauce/kg, 3.5 g of tomato sauce with refined olive oil/kg, and 0.25 g of sugar solved in water/kg	Healthy control	12 days	Tomato sauce enriched with refined olive oil can regulate lipid profile and soluble inflammatory biomarkers better than raw tomatoes or tomato sauces	2016 [51]

4	Double-blind controlled randomized trial	F = 79	56 ± 4	Genistein from soybeans (54 mg/day); estrogen/progestin therapy (1 mg/day); estrogen/progestin combined with norethisterone acetate (0.5 mg/day)	Healthy postmenopausal women	1 year	Genistein therapy improved endothelium function similar to estrogen/progestin regimen	2003 [52]

5	Double-blinded placebo-controlled crossover trial	93 (F = 51, M = 42)	25–65	Quercetin/day from fruits and vegetable sources (150 mg)	Overweight/obese	6 weeks	Reduced SBP and plasma oxidized LDL level with a high-CVD risk phenotype	2009 [53]

6	Double-blinded randomized placebo-controlled crossover trial	M = 31	35–51	Anthocyanins from fruits and vegetable sources (640 mg/day)	Prehypertensive	4 weeks	HDL-C and blood glucose were significantly higher after anthocyanin versus placebo treatment (*p* = 0.043 and *p* = 0.024, resp.)	2013 [54]

7	Randomized double-blind placebo-controlled clinical trial	105	20–60	One 350 mg whortleberry extract capsule every 8 hours	Hyperlipidemic	2 months	Lowered total-C (*p* < 0.001), TG (*p* = 0.002), and LDL-C (*p* = 0.002) but increased HDL-C levels (*p* < 0.001) compared with the placebo group at the endpoint	2014 [55]

8	Randomized double-blind placebo-controlled clinical trial	50	≥18	45 ± 2 mg of anthocyanin from *Vaccinium arctostaphylos* L. fruit extract	Hyperlipidemic	4 weeks	Significantly reduced total-C (*p* < 0.001), LDL-C (*p* = 0.004), TG (*p* < 0.001), and MDA (*p* = 0.013) compared to placebo	2014 [56]

9	Randomized controlled investigator blinded parallel-group trial, NCT00421499	44 (F = 24, M = 20)	56–73	Dark chocolate (6.3 g/day) containing 30 mg of polyphenols	Stage-1 hypertensive	18 weeks	Reduced mean (SD) SBP by 2.9 (1.6) mmHg (*p* < 0.001) and DBP by 1.9 (1.0) mmHg (*p* < 0.001)	2007 [57]

10	Randomized placebo-controlled single-blind crossover trial, NCT00538083	45 (F = 35, M = 10)	30–75	In phase 1, solid dark chocolate bar (containing 22 g cocoa powder) or a cocoa-free placebo bar. In phase 2, sugar-free cocoa (containing 22 g cocoa powder), sugared cocoa (containing 22 g cocoa powder), or a placebo	Healthy control	1 week	The acute ingestion of both solid dark chocolate and liquid cocoa improved endothelial function and lowered blood pressure in overweight adults	2008 [58]

*Diabetes mellitus (DM)*								

11	Open label randomized clinical trial	60	35–55	Standard metformin therapy with turmeric (2 g) supplements	Standard metformin treatment	4 weeks	Turmeric administered group significantly (*p* < 0.05) reduced the lipid peroxidation, MDA, inflammatory marker, CRP, HbA1c, and fasting blood glucose levels and enhanced the total antioxidant status	2015 [59]

12	Randomized double-blind controlled clinical trial	70	30–70	Powdered rhizome of ginger (1600 mg)	Wheat flour placebo (1600 mg)	12 weeks	Ginger significantly reduced the fasting plasma glucose, HbA1c, insulin, triglyceride, total cholesterol, CRP, and PGE2	2014 [60]

13	Randomized triple-blind controlled trial	M = 35	60 ± 11	Capsule of 350 mg of RES enriched grape extract (GE)	350 mg of capsules containing either maltodextrin (placebo) or RES lacking GE	12 months	RES enriched GE downregulated the proinflammatory cytokines expression via involving the inflammation-related miRs in circulating immune cells	2013 [61]

14	Randomized double-blind controlled trial	36 (F = 23, M = 13)	51.5 ± 10.0	FDS beverage of 2 cups (50 g of FDS is equivalent to 500 g of fresh strawberries)	Placebo powder with strawberry flavor	6 weeks	FDS significantly (*p* < 0.05) decreased the CRP levels and lipid peroxidation (mainly MDA) and increased the total antioxidant status	2013 [62]

15	Randomized double-blind controlled trial	64	38–65	Ginger supplementation	Placebo	8 weeks	Ginger supplementation significantly reduced the TNF-*α*, CRP, and IL-6 levels	2013 [63]

16	Randomized double-blind clinical trial	81	18–60	BSP at 10 g/day (*n* = 27) BSP at 5 g/day (*n* = 29)	Placebo	4 weeks	BSP significantly decreased the MDA, OSI, and oxidized low density lipoprotein cholesterol and increased the TAC	2011 [64]

17	Double-blinded randomized crossover trial	32 (M = 16, F = 16)	61.8 ± 6.4	GSE (600 mg/day)	Placebo	4 weeks	GSE significantly improved the inflammatory markers (mainly CRP), glycemia, and oxidative stress	2009 [65]

*Rheumatoid arthritis (RA)*								

18	Randomized single-blind pilot study	45 (F = 38, M = 7)	47.8 ± 8.6	Curcumin (500 mg) and diclofenac sodium (50 mg) alone or in combination	Diclofenac sodium (anti-inflammatory drug)	8 weeks	Curcumin alone proved to be the most effective (*p* < 0.05) and safest to attenuate RA complications	2012 [66]

19	Randomized double-blind placebo-controlled crossover study	20 (F = 19, M = 1)	52.1 ± 10.3	Quercetin (166 mg/capsule) + vitamin C (133 mg/capsule); *α*-lipoic acid (300 mg/capsule)	Placebo	6 weeks	No significant difference was found among the proinflammatory cytokines; however quercetin tended to reduce VAS of RA	2009 [67]

20	Randomized double-blind placebo-controlled study	105	55–75	Polyphenolic-rich olive extract	Placebo	8 weeks	Significant (*p* < 0.01) decrease of plasma homocysteine level (responsible for cardiovascular complications in RA) and notable reduction of CRP were observed	2007 [68]

F: female; M: male; RA: rheumatoid arthritis; VAS: visual analogue scale; NR: not reported; CRP: C-reactive protein; RES: resveratrol; GE: grape extract; miRs: microRNAs; FDS: freeze-dried strawberry; MDA: malondialdehyde; GSE: grape seed extract; BSP: broccoli sprouts powder; OSI: oxidative stress index; TAC: total antioxidant capacity; PGE2: prostaglandin E2; HbA1c: glycosylated hemoglobin; CAD: coronary artery disease; LDL-C: low density lipoprotein cholesterol; HDL-C: high density lipoprotein cholesterol; SBP: systolic blood pressure.

## Appendix

### Search Strategy

To find appropriate literature, the following terms were used in combination with Boolean operators (AND and OR).

*Search #1 (Fruits)*. (apple OR apricot OR applesauce OR avocado OR banana OR blackberries OR blueberries OR cantaloupe OR cherries OR dates OR dried fruits OR figs OR fruits OR fruit cocktail OR grapefruits OR grapes OR honeydew OR melon OR kiwi OR mango OR oranges OR papaya OR peaches OR pears OR pineapple OR plum OR raspberries OR strawberries OR watermelon).

*Search #2 (Vegetables)*. (amaranth OR Chinese spinach OR artichoke OR artichoke hearts OR asparagus OR baby corn OR bamboo shoots OR bean sprouts OR beets OR Brussels sprouts OR Broccoli OR green cabbage OR Chinese cabbage OR bok choy cabbage OR Italian beans OR green beans OR wax beans OR bean OR cabbage OR carrot OR cauliflower OR celery OR cucumber OR chayote OR daikon OR eggplant OR garlic OR ginger OR hearts of palm OR jicama OR kohlrabi OR leeks OR mushrooms OR onion OR okra OR pea pods OR peppers OR radish OR pepper OR black pepper OR red chills OR green chili OR rutabaga OR chicory OR escarole OR endive OR lettuce OR romaine OR arugula OR tomatoes OR Swiss chard OR spinach OR watercress OR turnip OR mustard OR collard OR sprouts OR radicchio OR peas OR turnips OR vegetables OR yard-long beans).

*Search #3 (Spices)*. (adobo seasoning OR allspice OR anise seed OR apple pie spice OR bay leaf OR cardamom seed OR cayenne OR chili peppers OR cinnamon OR cloves OR cumin OR curry powder OR fennel seed OR fenugreek seed OR five spice OR garam masala OR garlic OR ginger OR herb seasoning OR mace OR mustard OR nutmeg OR onion OR paprika OR peppercorns OR saffron OR sesame OR spice OR star anise OR thyme leaf OR turmeric).

*Search #4 (Phytochemicals)*. (antioxidants OR polyphenols OR carotenoids OR anthocyanins OR alkaloids OR glycosides OR saponins OR terpenes).

*Search #5*. (Search #1 OR Search #2 OR Search #3 OR Search #4).

*Search #6 (Degenerative Diseases and Factors)*. Cardiovascular diseases OR diabetes OR rheumatoid arthritis OR inflammation OR proinflammatory markers OR inflammatory OR oxidative stress OR reactive oxygen species.

*Search #7*. (Search #5) AND (Search #6).
